# Use of a real time continuous glucose monitoring system as an educational tool for patients with gestational diabetes

**DOI:** 10.1186/s13098-016-0161-5

**Published:** 2016-07-26

**Authors:** Eman Alfadhli, Eman Osman, Taghreed Basri

**Affiliations:** 1 Taibah University, Medina, Saudi Arabia; 2 Madina Maternity and Children Hospital, Medina, Saudi Arabia

**Keywords:** Gestational diabetes, Continuous glucose monitoring system, SMBG, Maternal glycemic control, Pregnancy outcomes

## Abstract

**Background:**

Women with gestational diabetes mellitus (GDM) are required to control their blood glucose shortly after GDM diagnosis to minimize adverse pregnancy outcomes. A real time-continuous glucose monitoring system (RT-CGMS) provides the patient with continuous information about the alterations in levels of the blood glucose. This visibility may empower the patient to modify her lifestyle and engage in therapeutic management. The aim of this study was to determine whether a single application of RT-CGMS to pregnant women shortly after GDM diagnosis is useful as an educational and motivational tool.

**Methods:**

This study was a prospective open label randomized controlled study conducted at Maternity and Children Hospital, Medina, Saudi Arabia. A total of 130 pregnant women with GDM were randomised to either blood glucose self-monitor alone (SMBG group) (n = 62) or in addition to SMBG, patients wore a Guardian^®^ REAL-Time Continuous Glucose Monitoring System (Medtronic MiniMed) once for 3–7 days, within 2 weeks of GDM diagnosis (RT-CGMS group) (n = 68). The primary outcomes were maternal glycemic control and pregnancy outcomes. Secondary outcomes were the changes in parameters of glucose variability, which includes mean sensor readings, standard deviation (SD) of blood glucose, and area under the curve for hyper and hypoglycaemia at the end of the RT-CGMS application.

**Results:**

HbA1c, mean fasting and postprandial glucose levels were similar in both groups at the end of the pregnancy. Pregnancy outcomes were comparable. However, there was significant improvement in the parameters of glucose variability on the last day of sensor application; both mean glucose and the SD of mean glycaemia were reduced significantly; P = 0.016 and P = 0.034, respectively. The area under the curve for hyper and hypoglycaemia were improved, however, the results were not statistically significant.

**Conclusion:**

Although a single application of RT-CGMS shortly after GDM diagnosis is helpful as an educational tool, it was not associated with improvement in glycemic control or pregnancy outcomes.

**Electronic supplementary material:**

The online version of this article (doi:10.1186/s13098-016-0161-5) contains supplementary material, which is available to authorized users.

## Background

Gestational diabetes mellitus, the most common complication of pregnancy, is usually diagnosed between 24 and 28 weeks of gestation. Within this narrow window of time, women with GDM are required to control their blood glucose swiftly after diagnosis to minimize adverse pregnancy outcomes. This requires disease awareness and self-control of blood glucose; however, these women usually have no experience and less knowledgeable in diabetes management and glycemia self-control.

A real time-Continuous Glucose Monitoring System provides the patient with continuous information about the alterations in the blood glucose levels throughout the day, which is immediately revealed to the patient, and helps the patient to understand how food, exercise, and insulin affect blood glucose [1]. This visibility may empower the patient to modify his/her lifestyle and engage in therapeutic management [2]. Nonetheless, RT-CGMS requires extra effort from patients and entails careful understanding on how to react according to the fluctuations of blood glucose levels. Using RT-CGMS, similar to the use of any other technology, requires sensible device utilization to obtain the optimal benefits from the system. The system also requires counseling support and encouragement by healthcare professionals. RT-CGMS studies in non-pregnant type 1 and type 2 diabetes patients reported its efficacy in improving glycaemic control [3–6]. In addition, intermittent use of retrospective CGM in pregnant women with pregestational diabetes [7] or GDM [8] was associated with improvement in blood glucose and pregnancy outcomes. However, Secher et al. [9] found that the use of intermittent RT-CGMS in pregnant women with well controlled pregestational diabetes did not improve glycemic control or pregnancy outcomes. There have been no studies evaluating the use of RT-CGMS in patients with GDM. The purpose of this study was to evaluate the impact of a single application RT-CGMS on maternal glycemic control and pregnancy outcomes for patients with GDM in comparison to the standard care and to assess its usefulness as an educational and motivational tool.

## Methods

A total of 130 pregnant women diagnosed with GDM attending antenatal services at Maternity and Children Hospital, Madinah, Saudi Arabia from October 2011 to June 2014 were included in the study. Women were eligible if they were diagnosed with GDM in the current pregnancy, had a singleton pregnancy, planned to give birth at the study hospital and were able to give written consent to participate. Exclusion criteria included pre-existing diabetes, multiple pregnancies, chronic diseases and drugs that might affect pregnancy outcome. The study was approved by the ethics committees of the Deanship of Scientific Research, Taibah University Medina, Saudi Arabia, and the Maternity and Children Hospital, Medina, Saudi Arabia. All women consented to participate after receiving a comprehensive explanation of the study.

Demographic data were obtained from all women during the first antenatal visit. Then, women were examined and weight, height, body mass index (BMI) and blood pressure were recorded. Oral glucose tolerance test were performed to all participants between 24 and 28 weeks of gestation. The diagnosis of GDM was based on the recommendations of the International Association of Diabetes in Pregnancy Study Groups (IADPSG). A specialist team consisting of obstetricians, internal medicine physicians, a diabetic educator, and a dietician followed the participants. Glucose values were evaluated weekly from self-monitoring of blood glucose (SMBG) four times per day, fasting and 2 h post meal, for the duration of the study. All participants were provided with a glucometer (Easy max). Glycemic targets were based on ADA recommendations as follows: fasting glucose ≤5.2 mmol/L (95 mg/dL); ≤7.8 mmol/L (140 mg/dL) 1-h post-meal; or ≤6.7 mmol/L (120 mg/dL) 2-h post-meal [8]. If the glucose values were persistently above the glycemic target on three or more occasions during the 1–2-week period, insulin was prescribed.

Patients were randomised to either blood glucose self-monitor alone (SMBG group) (*n* = 62) or in addition to SMBG, patients wore a Guardian^®^ REAL-Time Continuous Glucose Monitoring System (Medtronic MiniMed) once for 3–7 days, within 2 weeks of GDM diagnosis (RT-CGMS group) (*n* = 68). A random, computer-generated number list was used to assign patients to either the SMBG or RT-CGMS group.

Patients in the CGMS group were instructed to come to the research clinic in the morning after fasting for at least 4 h. Glucose sensors were inserted into the subcutaneous tissue at the upper buttock by the same trained nurses. Patients were instructed to continue performing SMBG four times daily and enter all blood glucose values directly into the RT-CGMS for calibration. The patients were also asked to record glucose values, time and contents of meals, insulin injections, exercise periods and symptomatic hypoglycaemic events in a logbook. Patients were allowed to look at their glucose value in the monitor and patients were encouraged to react appropriately. At the end of the monitoring period, the RT-CGMS results were downloaded into a computer and glucose profiles were generated. Subsequently, the resulting glucose profiles were reviewed by the researchers, and the treatment plan was adjusted accordingly. Daily mean sensor readings, SD of blood glucose, and the area under the curve for hyper and hypoglycaemia were noted from the RT-CGMS report. The values of these readings were compared between the first and last day of RT-CGMS application. Hypoglycaemia was defined as glucose values below 3.3 mmol/L (60 mg/dL), and hyperglycaemia was defined as glucose at or above 7.8 (140 mg/dL) mmol/L. Side effects from RT-CGMS were noted, e.g., skin irritation from the sensor. Acceptability of RT-CGMS by the patients was verified.

Patients in both groups were followed until delivery. Follow up frequency depended on the blood sugar control and week of gestation.

At the end of the pregnancy, the differences in diabetes control between the two groups were assessed by HbA1c, mean fasting and postprandial glucose levels, number of women needed insulin and total daily insulin dose. All measurements of serum glucose were performed by the glucose oxidase method. Formal laboratory HbA1c measurements were assessed by standardized HPLC.

Maternal and neonatal outcomes were collected after delivery from participants medical records and comparisons were made between the two groups.

Neonatal hypoglycaemia was defined as a blood glucose level below 2.2 mmol/L (40 mg/dL). An Apgar score >7 at 5 min was considered acceptable.

### Statistics

Statistical analyses were performed using SPSS software (v 16.0; SPSS Inc, Chicago, IL). Fisher’s exact test and χ^2^ analysis were performed to test for differences in the proportions of categorical variables between the two groups. Student’s *t* test (two-tailed) was used to determine the significance of differences between two continuous variables. A paired *t* test was used to determine the significance of differences in the parameters of glucose variability between the first day of sensor application and the last day. P < 0.05 was taken as the cut-off value for significance.

## Results

**Fig. 1:**
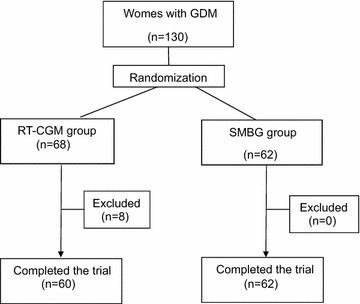
Subjects distribution

In total, 130 pregnant women were enrolled in the study. Eight patients were excluded from the RT-CGMS group as no glucose reports could be downloaded from the system. In two cases, this occurred because of sensor failures, in two other cases the electrodes fell of, and in four cases the patients did not perform calibration. Overall, 122 women completed the study, 60 in the RT-CGMS group and 62 in the SMBG group (Fig. 1).

Maternal baseline characteristics are shown in Table 1. There were no significant differences between the two groups. Baseline HbA1c and glucose levels at fasting, 1 and 2-h during OGTT were comparable.

**Table 1:** Baseline characteristics of the RT-CGMS and SMBG groups

Variable	SMBG group (*n* = 62)	RT-CGMS group (*n* = 60)	95 % CI	P value	
	Mean ± SD	Mean ± SD			
Age	34.15 ± 5.04	32.93 ± 5.70	−0.91 to 3.35	0.260	
Weight	76.34 ± 15.04	75.50 ± 19.08	−5.42 to 7.11	0.790	
BMI	32.11 ± 5.74	31.13 ± 7.53	−1.52 to 3.47	0.442	
Height	155.21 ± 7.84	155.94 ± 6.85	−3.47 to 2.01	0.601	
Systolic BP	120.72 ± 12.60	117.30 ± 13.01	−1.31 to 8.16	0.155	
Diastolic BP	67.63 ± 7.99	66.51 ± 7.30	−1.47 to 3.71	0.398	
Mean GA at GDM DX	23.95 ± 7.14	21.28 ± 6.26	−0.04 to 5.39	0.054	
Fasting OGTT (mmol/L)	5.01 ± 0.63	5.31 ± 1.06	−0.67 to 0.07	0.115	
1 h OGTT (mmol/L)	9.71 ± 2.05	10.41 ± 2.57	−1.70 to 0.29	0.166	
2 h OGTT (mmol/L)	8.72 ± 2.25	9.22 ± 3.11	−1.63 to 0.64	0.387	
HbA1c—mmol/mol (%)	41 ± 9 (5.9 ± 0.8)	38 ± 8 (5.6 ± 0.7)	−0.08 to 0.56	0.150	

	Number (%)	Number (%)	95 % CI	P value	Odd ratio
Multiparity^a^	40 (51.3)	38 (48.7)	0.18–1.11	0.063	0.45
History of recurrent abortion^b^	36 (56.2)	28 (43.8)	0.14–0.74	0.009	0.32
GDM in prior pregnancies	14 (23.3)	14 (25.0)	0.46–2.56	0.502	1.09
Acanthosis Nigrican	15 (27.8)	17 (30.4)	0.49–2.58	0.835	1.13
Family history of DM	41 (66.1)	44 (75.9)	0.72–3.57	0.315	0.82
History of preterm delivery	7 (11.5)	7 (12.3)	0.35–3.29	1.000	1.08
Glucosuria	4 (8.5)	9 (21.4)	0.83–10.35	0.085	2.93
History of still birth	4 (8.3)	7 (12.7)	0.43–5.85	0.537	1.60
History of neonatal deaths	2 (3.6)	2 (3.6)	0.36–7.35	1.000	1.00
History of large baby	5 (8.3)	7 (12.3)	0.16–2.14	0.552	0.59
History of malformed baby	7 (11.3)	4 (7.0)	0.29–3.62	0.533	1.01
History of gestational HTN	8 (13.8)	4 (7.0)	0.13–1.66	0.361	0.47
History of preeclampsia	6 (9.7)	3 (5.3)	0.12–2.17	0.494	0.51
History of medical illness	13 (21.7)	10 (17.5)	0.30–1.92	0.646	0.76
Previous SC	25 (64.1)	25 (67.6)	0.45–3.01	0.812	1.16

^a^Defined as two or more previous deliveries

^b^Defined as two or more previous abortions

### Real time-continuous glucose monitoring

The mean gestational age at the time of RT-CGMS’s application was 26 ± 5 weeks. The mean duration of the RT-CGMS registration period was 66.8 ± 2.3 h. As the levels of blood glucose were immediately revealed to the patient, 52.6 % reported some response to correct for hyper or hypoglycaemia, and 48 % received some modifications in their management plan after reviewing the RT-CGMS report by the research team.

RT-CGMS was generally well tolerated and there were no major side effects aside from mild erythema and skin irritation around the sensor’s insertion site. Indeed, the majority of patients (90 %) accepted the RT-CGMS. The reasons for not accepting the system were mainly due to technical challenges, in particular calibration, disparities between the RT-CGMS and SMBG readings, anxiety from continuous awareness of blood glucose levels, and mild local discomfort on laying down as the sensors were placed at the upper outer area of the buttocks.

### Parameters of glucose variability

**Fig. 2:**
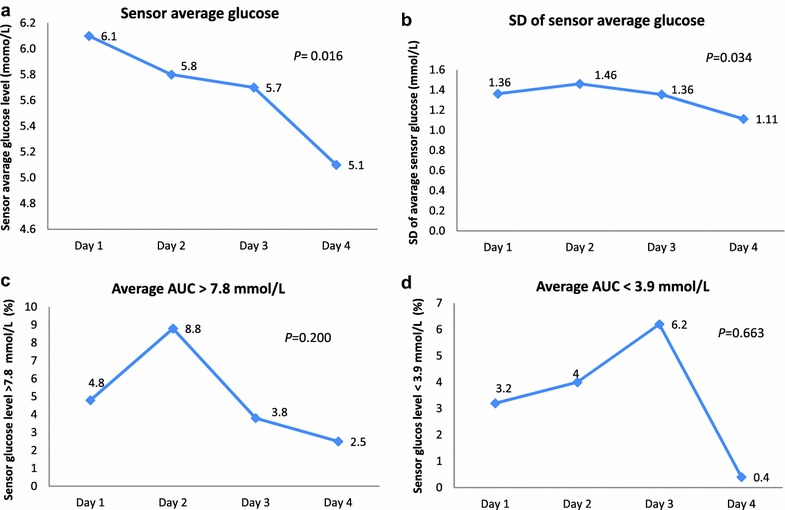
Parameters of glucose variability at the first and the last day of sensor application. **a** Sensor average glucose, **b** SD of the sensor average glucose, **c** average AUC > 7.8 mmol/L, and **d** average AUC < 3.9 mmol/L

There was significant improvement in the parameters of glucose variability by the last day of sensor application (see CGMS’s report for one patient, Additional file 1: Figure S1). Both mean sensor glucose and SD of the sensor glucose were reduced significantly, P = 0.016 and P = 0.034, respectively (Fig. 2a, b). Although, the area under the curve for both hyper and hypoglycaemia were improved by the last day of sensor application, the results were not statistically significant (Fig. 2c, d).

### Glycemic control

HbA1c, mean fasting and postprandial glucose level were comparable between the two groups at the end of the pregnancy. In addition, there were no significant differences in the number of women who required insulin therapy or the total daily insulin dose between both groups, Table 2.

**Table 2:** Glycemic and pregnancy outcomes

Outcome variable	SMBG group	CGMS group	P value	Odd ratio	95 % CI
Glycemic outcomes					
Fasting glucose	4.99 ± 1.01	4.71 ± 0.68	0.092	–	−0.272 to 0.3700
Postprandial glucose	6.27 ± 1.36	5.72 ± 1.59	0.057	–	−0.0169 to 1.099
HbA1c at the end of the pregnancy—mmol/mol (%)	43 ± 5 (6.1 ± 0.4)	39 ± 8 (5.70 ± 0.7)	0.168	–	−0.1955 to 1.0098
Number of women needed insulin (%)	18 (11.16)	19 (11.4)	0.896	1.056	0.467 to 2.384
Total daily insulin dose (units)	29.83 ± 43.83	20.67 ± 18.03	0.410	–	−13.103 to 31.436
Maternal outcomes					
Polyhydramnios	0	0			
GA at delivery (week)	38.17 ± 1.63	38.35 ± 2.026	0.627	–	−0.898 to 0.544
Preterm delivery	9.5	16.3	0.373	1.854	0.516 to 6.659
PROM	3.9	8.3	0.427	2.227	0.389 to 12.760
Induction of labour	13.0	14.6	1.000	1.146	0.371 to 3.543
SC delivery	49.1	55.1	0.562	1.273	0.588 to 2.755
Lacerations	2.4	6.4	0.620	2.727	0.273 to 27.293
Shoulder dystocia	0	0			
ICU admission	9.8	4.3	0.415	0.420	0.073 to 2.426
Neonatal outcomes					
Abortion	0	2.0	1.000	0.527	0.434 to 0.641
Stillbirth	2.1	0.0	0.457	0.452	0.366 to 0.558
Neonatal death	1.6	3.4	0.613	2.14	0.149 to 20.76
Foetal injury	0.0	0.0	–	–	–
Apgar score <7 at 5 min	16.7	10.0	0.503	0.556	0.143 to 2.153
Birth weight (g)	3056 ± 564	2870 ± 610	0.130	–	−0.055 to 0.428
Macrosomia	2.4	0	0.488	0.482	0.387 to 0.601
Low birth weight	9.5	22.2	0.147	2.714	0.780 to 9.447
Hypoglycaemia	12.8	16.7	0.758	1.360	0.393 to 4.704
Hyperbilirubinemia	10.3	14.6	0.738	1.500	0.389 to 5.781
Congenital malformation	10.3	4.8	0.421	0.075	2.535 to 2.886
RDS	10.6	7.1	0.717	0.646	0.145 to 2.885
NICU admission	30.0	34.8	0.653	1.244	0.502 to 3.087

Results are given as the percentages (%) or mean ± SD. Preterm delivery refers to delivery before 37 weeks of gestation; PROM, premature ruptures of membranes; macrosomia, defined as birth weight of 4000 g or more; low birth weight defined as birth weight less than 2500 g

*RDS* respiratory distress syndrome

### Pregnancy outcomes

Of the 122 pregnancies, there was one miscarriage and 121 live births. Five infants had congenital malformations, with three cardiovascular malformations in the SMBG group and two in the RT-CGM group (one cardiovascular and one anus malformation).

Approximately half of the deliveries were by caesarean section with no differences between the two groups. Similarly, there were no differences in the gestational age at deliveries, birth weight, prevalence of macrosomia and neonatal hypoglycaemia.

There were no statistically significant differences in the other maternal and neonatal outcomes between the two groups, Table 2.

## Discussion

To our knowledge, this study is the first prospective study to examine the use of real time CGMS in patients with GDM. We found that a single application of RT-CGMS for pregnant women shortly after GDM diagnosis is useful as an educational and motivational tool. It gives the pregnant woman insights on the effect of food, exercise, and insulin on blood glucose, which helps in modifying the patient’s diet and exercise practices. In line with this finding, previous studies have shown the usefulness of RT-CGMS as an educational and a motivational tool for poorly controlled type 1 [3, 4] and type 2 diabetes.[6].

Blood glucose variability has a significant impact on the quality of life and appears to be associated with the development of diabetes complications [11]. A reduction in glycemic variability alone was suggested to improve diabetes outcomes even with no improvement in HbA1c [11]. In the present study, there was significant improvement in blood glucose variability with a single application of RT-CGMS to GDM patients. This is in line with previous studies that used RT-CGMS in type 1 and type 2 diabetic patients [4–12] and other studies that used retrospective CGMS in patients with GDM [8]. In the current study, mean glycaemia, SD of mean glycaemia, and the area under the curve for both hyper and hypoglycaemia were used to assess the glycemic variability, and we found significant improvement in the mean glycaemia and the SD of mean glycaemia by the last day of RT-CGMS application. The area under the curve for both hyper and hypoglycaemia were also improved; however, the results were not statistically significant.

Despite the improvements in glycemic variability during RT-CGMS application, there were no differences in glycemic control between the RT-CGMS and the standard antenatal care group at the end of pregnancy. This is in agreement with a study by Yu et al. in which the researchers found significant improvements in glycemic variability; however, the mean blood glucose levels were similar between the CGM and the routine care group and the researchers study did not evaluate HbA1c at the end of the pregnancy [8]. In addition Secher et al. [9] did not find improvement in glycemic control when applying RT-CGMS intermittently to pregnant women with well-controlled type 1 and type 2 diabetes. This is also in line with a study on well-controlled non pregnant diabetic patients in whom there was no additional improvement in HbA1c with RT-CGMS application [13].

On the other hand, some studies have demonstrated the effectiveness of RT-CGMS on glycemic control in non-pregnant patients with type 1 or type 2 diabetes [4–12] as well as the uses of intermittent retrospective CGM in patients with pregestational diabetes [7]. In those studies, participants had higher HbA1c at baseline compared to the participants in our study and other studies with negative results. This might explains the disagreement between the findings. As the level of HbA1c tends to decrease in pregnancy due to the rise in red cell mass and red blood cell turnover, the use of HbA1c to assess glycemic control in GDM women with low HbA1c levels at the initial visit may not be useful [14]. The mean baseline HbA1c in our cohort of women was 40 mmol/mol [range 31–49], (5.8 %) [range 5–6.7], which might explain our negative results on HbA1c.

Our finding of the lack of effectiveness of RT-CGMS on pregnancy outcomes was in agreement with a study by Secher et al. in which RT-CGMS was used in pregnant women with well-controlled type 1 and type 2 diabetes [9], and is in contrast to the findings from the other studies that confirmed the improvement in pregnancy outcomes when using retrospective CGM in patients with pregestational diabetes [7] or GDM [8]. Murphy et al. [7] found lower birth weight and lower risk of macrosomia when using retrospective CGM intermittently in patients with pregestational diabetes, and Yu et al. [8] found less risk of preeclampsia and caesarean deliveries, lower birth weight, and less neonatal complications when using retrospective CGM intermittently on GDM patients.

The reason for the disagreement between the studies on the effect of CGMS on glycemic control and pregnancy outcomes can be attributed to several factors, including baseline glycemic control, size of the study sample, duration of CGM application, and patient selection. Well-controlled diabetic patients at baseline may have no further benefits from using CGM. The smaller the study sample size the lower the power for finding significant results. Indeed, we found lower levels of HbA1c and mean fasting and postprandial glucose along with lower doses of insulin in the RT-CGMS group than in the standard group; however, the results did not reach statistical significance. In addition, longer application of RT-CGMS may give better results than shorter usage, as there is a learning curve for women using the system. Finally, patient selection is an extremely important factor. Without a doubt, the key issue for RT-CGMS success is selecting appropriate patients. Highly motivated patients who have an interest in using this technology and are enthusiastic to react accordingly will benefit most from using the system. If the patient is reluctant to respond to the data delivered by RT-CGMS, the system will be useless. This was illustrated by a study that applied RT-CGMS for one month to T1DM adolescent patients. Although they found significant improvement in glycemic control, the use of this technology was not efficient for those with a very high HbA1c [HbA1c >86 mmol/mol (10 %)] at baseline, in whom compliance and self-motivation are the main concerns [4]. Therefore, we still believe in the favourable effects of RT-CGMS in patients with diabetes if applied to the right person.

RT-CGMS was accepted by most of the participants, and the reasons for non- acceptance were technical challenges, such as calibration and frustration with sensor alarms, anxiety from continuous awareness of blood glucose level, skin reaction, and disparities between RT-CGMS and SMBG readings. Admittedly, a significant number of our women had readings in the hypoglycaemic range during the first day of RT-CGMS application, which conflicted with the SMBG reading, a finding that has been reported previously by Secher et al. [9]. This indicates that there is still a need for improvement in the RT-CGMS accuracy in the hypoglycaemic range.

Limitations to the current study include single use of RT-CGMS, which might be the reason for non-improvement in glycemic control or pregnancy outcomes. However, our aim was to test the efficacy of a single application of RT-CGMS in GDM because it is more convenient and acceptable to the patients and less costly. Another limitation of our study is the relatively small sample size, which may have limited the power to detect differences in glycemic control or in pregnancy outcomes. A larger clinical trial with a selection of highly motivated patients is recommended to evaluate the effectiveness of RT-CGMS in pregnant women with GDM. In addition, cost effectiveness studies assessing the application of RT-CGMS in these women versus standard care is also needed.

## Conclusions

A single application of RT-CGMS is useful as an educational and a motivational tool for patients with GDM and helps in improving blood glucose variability. However, these changes are not coupled with improvement in HbA1c and pregnancy outcomes. Using RT-CGMS, similar to any other technology, requires sensible utilization of the device to obtain the greatest benefit from the system and the key factor in achieving success is selecting appropriate patients.

## Additional file

10.1186/s13098-016-0161-5 CGM report for one patient.
