# Supplementary figures and images for: Use of a real time continuous glucose monitoring system as an educational tool for patients with gestational diabetes
Source: Diabetol Metab Syndr. 2016 Jul 26;8:48. doi: 10.1186/s13098-016-0161-5 (PMC4962392; doi:10.1186/s13098-016-0161-5)

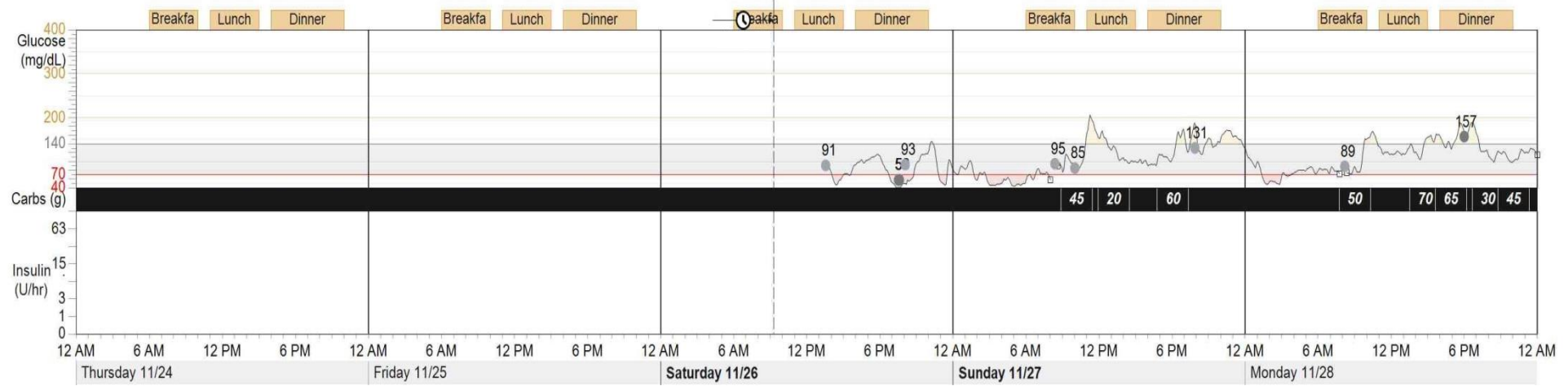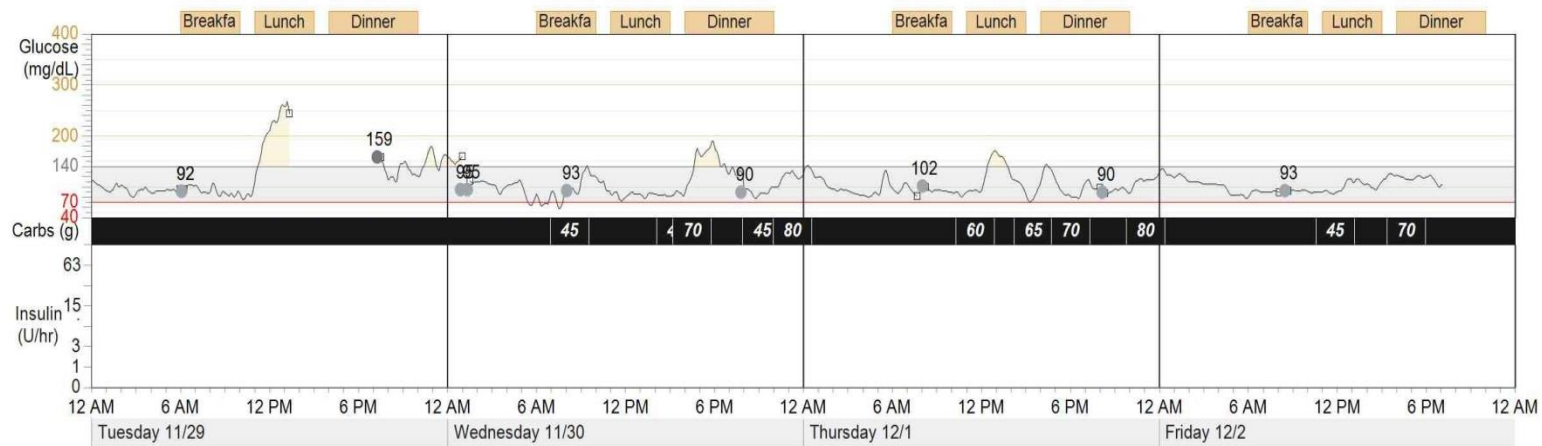

Supplement: Supplementary file 1 — 10.1186/s13098-016-0161-5 CGM report for one patient. [file 13098_2016_161_MOESM1_ESM.pdf]
